# Corrigendum: Evaluation of the Results of Septum Extension Batten Graft in Patients Referred for Septorhinoplasty: São Paulo, October 23, 2023

**DOI:** 10.1055/s-0043-1776756

**Published:** 2023-11-14

Dear readers,

In the Article *Evaluation of the Results of Septum Extension Batten Graft in Patients Referred for Septorhinoplasty* (Int Arch Otorhinolaryngol 2023;27.4:602–607. DOI: 10.1055/s-0043-1768211 ), published online in *Int Arch Otorhinolaryngol* in October 2023.

**Where it reads**

Hamidreza Hosnani ^1^ Shahin Bastaninez ^1^ Amirbahador Golchin ^1^ Hamed Givzadeh ^1^

**It should read**

Hamidreza Hosnani ^1^ Shahin Bastaninejad ^1^ Amirbahador Golchin ^1^ Hamed Givzadeh ^1^

